# Supplementary material for: Study on the methodology of emergency decision-making for water transfer project contingencies: A case-based reasoning and regret theory approach
Source: PLoS One. 2024 Mar 21;19(3):e0300272. doi: 10.1371/journal.pone.0300272 (PMC10956882; doi:10.1371/journal.pone.0300272)
Supplement: Attachment — Submitted filename: Review Comments of manuscript PONE-D-23-38121.docx [file pone.0300272.s001.docx]

**Manuscript Number: PONE-D-23-38121**

**Title:** **Study on the Methodology of Emergency Decision-Making for Water Transfer Project Contingencies: A Case-Based Reasoning and Regret Theory Approach**

**Comment 1**: In the introduction part the objectives of importance of the study have to clearly stated

**Comment 2**: the overall significance of the study needs to clearly state.

**Comment 3**: the introduction part is very bulky. It needs to make it clear by modifying it. The research gap and objectives have to clearly provide in this part.

**Comment 4:** In introduction part on line 37 ‘According to statistics’ it needs to provide the reference for justification.

**Comment 5:** also in introduction part on line 39 ‘As a major water transfer project in China, the South-to-North Water Diversion Project has 40 transferred over 40 billion cubic meters of water’ it needs justification

**Comment 6:** Are you sure you are following the guideline manuscript preparation of PLOS ONE? Why include Literature review? Let check it.

**Comment 7:** In methodology part, line 192 where is the source of equation? Before providing the equation you have to clearly discuss how the equation used.

**Comment 8:** On page 235, why you use future tense ‘solution to the above problem will be addressed’’

**Comment 8:** on line 573 you mentioned to use both TOPSIS and fuzzy comprehensive evaluation methods it needs to clearly describe the difference between both methods and which one is more preferable for your research?

**Comment 10:** some references are very old and out dated (i.e. *Chen, S.-M.; Tan, J.-M. Handling multicriteria fuzzy decision-making problems based on vague set theory. Fuzzy Sets and Systems 1994, 67, 163-172,doi:https://doi.org/10.1016/0165-0114(94)90084-1)*

Reviewer Name: Siraj Abduro Abdulahi

Email: [sirajab25@gmail.com](mailto:sirajab25@gmail.com)
