# Supplementary material for: Study on the methodology of emergency decision-making for water transfer project contingencies: A case-based reasoning and regret theory approach
Source: PLoS One. 2024 Mar 21;19(3):e0300272. doi: 10.1371/journal.pone.0300272 (PMC10956882; doi:10.1371/journal.pone.0300272)
Supplement: Attachment — Submitted filename: Authors Reply to the Review Report (Reviewer 2).docx [file pone.0300272.s002.docx]

On behalf of all the contributing authors, I would like to express our sincere appreciations of your letter and reviewers’ constructive comments concerning our article entitled“Study on the Methodology of Emergency Decision-Making for Water Transfer Project Contingencies: A Case-Based Reasoning and Regret Theory Approach”(Manuscript No: PONE-S-23-50080). These comments are all valuable and helpful for improving our article. According to your comments, we have made extensive modifications to our manuscript. In this revised version, changes to our manuscript were all highlighted within the document by using yellow-colored text. The following are specific responses to each question.

**Author's Reply to the Review Report (Reviewer 2)**

We sincerely appreciate the valuable feedback from editor and reviewers, which we use to improve the quality of the manuscript. The reviewers' comments are presented in black, bolded font, and specific questions have been numbered. Our recoveries are given in normal font, with changes/additions to the manuscript in yellow text.

**Comment 1: The abstract needs improvement to clarify the main topic of this paper.**

We feel great thanks for your professional review work on our manuscript. Here is our improved abstract.

**Abstract:** In order to tackle the global water imbalance problem, a multitude of inter-basin water transfer projects have been built worldwide in recent decades. Nevertheless, given the complexity and safety challenges associated with project operation, effective emergency decision-making is crucial for addressing unforeseen incidents. Hence, this research has developed a two-stage emergency decision-making framework to tackle the uncertainty in the development trends of emergencies in inter-basin water transfer projects. (1) The first stage mainly utilizes case-based reasoning techniques to extract historical case information and disposal plans for inter-basin water transfer projects. Subsequently, a holistic similarity model is built by employing structural similarity and local attribute similarity algorithms to identify highly similar historical cases. (2) The second stage involves the optimization and adjustment of decision-making plans based on the dynamic evolution characteristics of emergencies. It utilizes the theory of decision-makers' regret psychology and combines with practical case studies to verify the scientific rationality of the method. This enables it to achieve effective multidimensional expression and rapid matching of scenarios, satisfying the decision-making requirements of "scenario response". Finally, this study compares the results obtained from this method with those computed using the traditional TOPSIS method and fuzzy comprehensive evaluation method, further validating its feasibility and effectiveness. In practice, this method can provide effective support for decision-makers' work.

**Comment 2: Some keywords are missing.**

We are sincerely grateful for your constructive comments on our manuscript. Thanks for your reminder. Here are our improved keywords.

**Keywords:** Inter-basin water transfer projects; Emergency decision-making for emergencies; Case-based reasoning; Regret theory; TOPSIS method; Fuzzy integrated evaluation method

**Comment 3: Please revise the whole paper in terms of punctuation, especially for the equations. Some commas and full stops are missing.**

We are sincerely grateful for your constructive comments on our manuscript. We did our best to correct some punctuation in the manuscript, as well as a few careless errors, and would appreciate your comments.

**Comment 4: The introduction needs to be improved and arranged properly with paragraphs to separate the previous knowledge, the research gaps, a clear input you have made and the current paper arrangement.**

We feel great thanks for your professional review work on our manuscript. We have made a lot of changes to the introduction part. We have divided the introduction into five paragraphs. The first paragraph introduces the concepts and benefits associated with inter-basin water transfer projects and explains the background of the study. The second paragraph analyses the serious problems such as emergencies that are prone to occur in the operation of the current inter-basin water transfer project, and leads to the research purpose of this paper, which is the study of emergency decision-making and disposal of emergencies in the inter-basin water transfer project. The third paragraph explains the research methodology adopted in this paper and illustrates the current research gaps as well as the significance of this paper. The fourth paragraph briefly describes the main contributions of this study. Finally, the fifth paragraph briefly describes the arrangement of the parts of the current article. Here is the revised introduction, and we hope to get your comments on it.

**Comment 5: Please cite some recent relevant papers to improve the introduction part.**

We feel great thanks for your professional review work on our manuscript. We think this is an excellent suggestion. Based on your comments, we have made corrections to make the article more coherent and persuasive by including relevant papers from recent years in the introduction part.

**Comment 6: An improvement in the quality of the figures is needed.**

We feel great thanks for your professional review work on our manuscript. We have modified **Figure 2.,** **Figure 3.** and **Figure 4.** as much as possible in the newly submitted manuscript. I wonder if we can get your satisfaction.

**Comment 7: Read the whole manuscript and try to remove the passive voice misuse and typo mistakes in some spots.**

We feel great thanks for your professional review work on our manuscript. We feel sorry for our carelessness and have tried our best to polish the language in the revised manuscript. We appreciate for your warm work earnestly and hope that the correction will meet with approval.
